# Chorological maps for the main European woody species

**DOI:** 10.1016/j.dib.2017.05.007

**Published:** 2017-05-06

**Authors:** Giovanni Caudullo, Erik Welk, Jesús San-Miguel-Ayanz

**Affiliations:** a Engineering Ingegneria Informatica S.p.A., Via San Martino della Battaglia 56, Rome I-21018, Italy; b Institute for Biology, Martin-Luther- University Halle-Wittenberg, Am Kirchtor 1, 06108 Halle (Saale), Germany; c German Centre for Integrative Biodiversity Research (iDiv) Halle-Jena-Leipzig, Deutscher Platz 5e, 04103 Leipzig, Germany; d European Commission, Joint Research Centre (JRC), Directorate for Space, Security and Migration, Disaster Risk Management Unit, Via E. Fermi 2749, I-21027 Ispra, VA, Italy

**Keywords:** Species range, Botany, Tree, Shrub, Distribution

## Abstract

A novel chorological data compilation for the main European tree and shrub species is presented. This dataset was produced by combining numerous and heterogeneous data collected from 20th century atlas monographs providing complete species distribution maps, and from more recent national to regional atlases, occurrence geodatabases and scientific literature. The dataset is composed of numerous species distribution maps available in geographical information system (GIS) format, created by compiling, evaluating and synthesizing data of all collected sources. The geometry of the individual datasets describes contiguous large areas of occupancy of each species as polygons and fragmented or isolated occurrences as points. Since this geodatabase is intended to provide a synthetic continental-scale overview of the species ranges, the maps represent the species’ general chorology and the presence/absence information should not be considered absolute in terms of geolocation. Errors and imprecisions arising from the interpretation and digitalization processes are likely to occur, especially in those areas where detailed information is scarce. As new information sources become available, these will be used to address current data gaps, implement corrections and updates of the chorology dataset as well as expanding it to comprise additional species.

**Specifications Table**

**Table:** 

Subject area	Botany
More specific subject area	Geobotany, Floristics, Chorology
Type of data	Vector ESRI shapefiles, text file
How data was acquired	Geographic information system (GIS)
Data format	Analysed
Experimental factors	Printed maps in published monographs were scanned/digitalized, then all images were geo-referenced with GIS software.
Experimental features	Geo-referenced partial and complete chorological maps and species occurrence geodatabases were overlaid, compared and merged to create new updated distribution range maps in geographic digital format.
Data source location	Worldwide
Data accessibility	On Mendeley Data repository: http://data.mendeley.com/datasets/hr5h2hcgg4

**Value of the data**

• The data provides a set of distribution ranges of European tree and shrub species in a geographic digital format, which has been compiled by expert comparisons and analyses of numerous and heterogeneous sources.
• The data are stored in ESRI shapefile format and can be easily handled in GIS software for mapping the species distribution ranges, presenting and describing synthetically where the species generally occur.
• While currently limited in areas with scarce information, the data will be updated as soon as new sources become available.
• These new dataset can contribute significantly to research in the fields of ecology, forest genetics, biodiversity, phytosanitary and pest control activities, among others.

## 1 Data

The data are organized as a set of ESRI shapefiles (*.shp, *.shx, *.dbf, *.prj files) mapping the distribution ranges of the main European tree and shrub species. For each species and in some cases subspecies, one or more shapefiles have been created containing: a) polygon features (name suffix “plg”), which define continuous areas of occupancy of the species range and b) point features (name suffix “pnt”), which identify more fragmented and isolated populations. For species with reported synanthropic occurrences outside the natural range, an additional point and/or polygon shapefile has also been created (suffix “syn”). The polygon borders delimiting the range have been generalized across the mainland and sea boundaries. Clipping to a specific coastline has been avoided, as this can vary considerably in its geometry depending on scale and precision of the respective source. This offers the possibility to mask sea areas, or to clip and extract the species’ terrestrial range parts using GIS data layers of the users’ choice. Finally, an accompanying text document is included with the data, which provides more details on methodology and a list of all mapped species with related file names, taxonomical delimitation of the mapped species and references used to compile the respective chorological dataset.

**Fig. 1:**
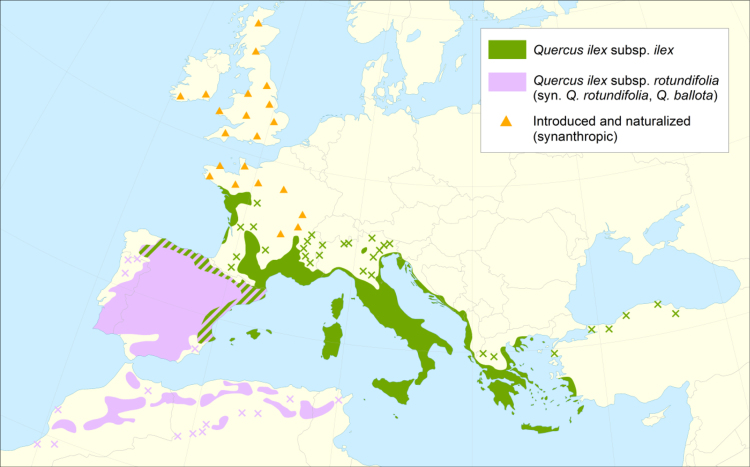
Example of a chorological map of the holm oak (*Quercus ilex* L.). The distributions ranges of the two subspecies are shown in different colours; where both subspecies occur, alternating colour strips are used. Isolated populations and synanthropic areas are shown as point features and symbolized in the map as crosses and triangles, respectively.

## 2 Experimental design, materials and methods

The information sources for compiling distribution data were numerous and heterogeneous. Base chorological maps covering the entire range of species were acquired principally from seminal monographs published in the second half of last century [1], [2], [3], [4]. Continental maps were found in historical and in more recent publications providing species distribution information over Europe [5], [6], [7], North America [8], [9] and Asia [10], [11], [12]. If available, point and polygon geodatabases supplying geo-localized species presence were also used [13], [14], [15]. Finally, species presence from country to regional level was included as documented in specific textbooks, journal papers and websites.

The chorological maps found in printed publications were digitalized and converted into high resolution images, successively imported and geo-referenced into GIS software and finally overlaid to the collected point/polygon geodatabases. Additionally, a digital elevation model (DEM) was used as background information on the orography of the mapped areas. By comparing, evaluating and synthesizing the information of all different sources, continuous areas of occupancy of the species were drawn as polygons. Single or small concentrations of occurrence locations separated from the main continuity of the species range were considered as isolated populations and digitalized as point features instead of polygons. For those plant species occurring also outside the native ranges, the distribution area was digitalized separately as introduced and naturalized range (synanthropic).

Since the maps aim at representing the species general chorology at continental scale, providing a synthetic overview of distribution range, the mapped boundaries should not be considered as precise and sharp limits where the species is definitely present or absent, particularly at local level. Indeed, the first version of this dataset was created for the European Atlas of Forest Tree Species [16] to concisely outline the distribution ranges of described species, complementing information on the species biology and ecology. Errors and imprecision are partly inevitable, due to various causes, such as the quality of the original source, the geo-referencing procedure, the interpretation and the comparison of the sources in the same area and finally due to the limited precision of the manual digitalization process of the range borders (Fig. 1).
